# Food Marketing Targeting Youth and Families: What Do We Know about Stores Where Moms Actually Shop?

**DOI:** 10.1155/2013/674181

**Published:** 2013-09-16

**Authors:** Diana S. Grigsby-Toussaint, Mary R. Rooney

**Affiliations:** ^1^Division of Nutritional Sciences, Department of Kinesiology and Community Health, University of Illinois at Urbana Champaign, 1206 S. Fourth Street, 2019 Huff Hall, Champaign, IL 61821, USA; ^2^Department of Kinesiology and Community Health, University of Illinois at Urbana Champaign, IL 61821, USA

## Abstract

Although efforts are underway to examine marketing that targets the youth and families in the retail food store environment, few studies have specifically focused on stores that families identify as their primary sites for food shopping. Between November 2011 and April 2012, we examined the frequency and types of marketing techniques of 114 packaged and nonpackaged items in 24 food stores that mothers of young children in Champaign County, IL, said they commonly frequented. Chi-square tests were used to determine whether significant differences existed between items with regard to marketing by store type, store food-assistance-program acceptance (i.e., WIC), and claims. Overall, stores accepting WIC and convenience stores had higher frequencies of marketing compared to non-WIC and grocery stores. Fruits and vegetables had the lowest frequency of any marketing claim, while salty snacks and soda had the highest frequency of marketing claims. Nutrition claims were the most common across all items, followed by taste, suggested use, fun, and convenience. Television tie-ins and cartoons were observed more often than movie tie-ins and giveaways. Our results suggest an opportunity to promote healthful items more efficiently by focusing efforts on stores where mothers actually shop.

## 1. Introduction

The obesity epidemic in the United States is a major public health problem for children [1, 2]. Recent estimates from the National Health and Nutrition Examination Survey indicate that approximately 16.9% of children are considered obese [1]. Curbing the onset of overweight and obesity in childhood is of particular concern, given the attendant comorbidities such as sleep apnea and type 2 diabetes, which may affect quality of life and increase the long-term burden on the US health care system [3]. Although individual-level factors like genetics clearly play a role in weight imbalance and the subsequent onset of obesity [4], emerging empirical evidence suggests that aspects of the food environment influence a major risk factor for obesity, namely, dietary behavior [5–8].

Broadly defined, the food environment refers to the availability and accessibility of food in multiple spaces (e.g., community food stores and schools); it also relates to the policies that influence the availability of healthy foods, product placement, and marketing within the stores that influence purchasing behaviors and, ultimately, eating patterns [9, 10]. In-store food marketing, in particular, is gaining prominence as a focus for curbing the obesity epidemic among children and families [11–15].

There are several reasons for this trend. First, there is growing evidence that exposure to food marketing is associated with increased risk for childhood obesity [16]. Marketing of energy-dense foods can influence dietary consumption patterns and increase obesity risk [17–19]. Second, food and beverage companies spend a great deal of money promoting their products in supermarkets; for instance, a 2002 study showed that these companies spent even more on in-store promotions than on television advertising ($234 billion versus $212 billion) [20]. Third, as the point of purchase for food for many families, retail food stores provide a critical location for interventions to promote healthy eating [11, 21]. For example, a recent grocery store intervention by Holmes et al. [21] found that the use of a kiosk promoting fruits and vegetables and healthy snacks resulted in a higher proportion of sales for these items during the intervention period. In addition, in-store marketing techniques, such as manipulating shelf space, have long been recognized as a way to improve the sales of certain food items [22]. Finally, evidence suggests that children are increasingly spending more time shopping with their parents and influencing the purchase of foods in the retail food store environment [23]. Unfortunately, most children typically ask their parents to buy empty calories, such as candy or other sweets [23, 24]. Taken together, the research shows that retail food stores provide an important setting for population-based interventions using food marketing to encourage healthy dietary behaviors among children and families.

Although several efforts are underway to examine marketing targeting youth and families in retail food stores, most studies have focused on unhealthy packaged products and have paid only limited attention to specific claims used to market typically healthier nonpackaged items like fruits and vegetables [15, 25–28]. This focus may be a function of expenditures by food and beverage companies on in-store food marketing: the Federal Trade Commission estimated that in 2009, of the $113 million spent on in-store marketing to children and teens (ages 2–11 and 12–17, resp.), restaurant foods ($27 million), snack foods ($12 million), and breakfast cereals ($10 million) were the 3 most frequently marketed, while fruits and vegetables had the lowest frequency, with $722,000 being spent on marketing to children and $0 to teens [18]. 

Another limitation of studies examining the impact of the retail food store environment on risk factors for chronic conditions is that they assess a random sample of all the stores in an area, rather than focusing on stores where residents actually shop. A study by Chaix et al. [29] that examined the weight and self-reported food shopping behaviors of participants found that stores in the participants' residential areas accounted for less than 12% of the stores they actually shopped at. 

We wanted our study to accurately reflect the food environment of mothers of young children in our study area, so we selected stores for our analysis based on their reported stores of choice. We also compared types and amount of packaging-related marketing observed in the stores where mothers actually shopped with data from a previous study that evaluated all stores in the study area. 

Furthermore, we sought to investigate the frequency of marketing techniques that targeted children and families in these retail food stores. We focused specifically on product packaging, examining the presence of claims and the use of cartoon characters, movie tie-ins, television tie-ins, and giveaways (e.g., toys). Claims were of interest due to a population-based analysis indicating that 43.8% of adults and 54.3% of women examined claims on food packaging in order to make purchases [30]. Additionally, preliminary analysis of qualitative interviews with women in our study area suggests the influence of marketing on purchases in food stores, as evidenced by the following quote:

*“Sometime(s) marketing influence(s) what I buy because my son asks me for products he watched on TV commercials.” *

The presence of cartoon characters on food packaging has been shown to influence children's preferences for specific foods [31], and movie tie-ins, television tie-ins, and giveaways have been used by food and beverage companies to improve product appeal to children [20]. We specifically examined more healthful items such as fruits and vegetables in addition to items typically marketed to children and families, like cereals, juice, and salty snacks. We looked for differences in how various items within stores were marketed, as well as how the same items were marketed in different kinds of stores. 

## 2. Methods

### 2.1. Setting

The cities of Champaign and Urbana, IL, located in the midwestern United States, were selected as the target areas for this study. Both cities are part of the sampling frame for two ongoing studies of mothers of young children currently being conducted by the lead author of this study. One ongoing study focuses on childhood obesity with a subset of mothers of preschool aged children recruited from child care centers (*n* = 165) in central Illinois [32], and the second study, as yet unpublished, examines the utilization of the food environment of participants in the Supplemental Nutrition Program for Women, Infants, and Children (WIC) in Champaign County, IL (*n* = 60) (information available on request).

As of 2010, the combined population of Champaign and Urbana was 122,305 residents, of which 21,183 were families [33]. The median income for Champaign and Urbana was $34,263, which is only 63% of the median income for the state of Illinois, at $53,966 [33]. Across both cities, Whites, Blacks, and Hispanics comprise 64%, 15.9%, and 5.79% of the population, respectively [34].

### 2.2. Sample

Our sample included 25 retail food stores identified as being commonly patronized by mothers of young children in Champaign and Urbana, IL. These stores were identified by surveying 225 mothers of young children for the names and addresses of the top five stores they typically frequented. Mothers surveyed from the WIC program had lower levels of education (38.2% had less than a high school degree) than non-WIC participants. Approximately 51.8% of the WIC participants were non-Hispanic White. Mothers surveyed from preschools in the study area were slightly more likely to be non-Hispanic White; 66.9% of these women had higher levels of education, and 53.8% were college graduates.

We used the survey results to select a mix of supercenters, small grocery stores, and convenience stores. Our selection process also took into account directional deviational ellipses that we created using the spatial statistics toolbox in ArcGIS 9.3 [35] to determine how many participants were shopping at a particular store, as well as how far they were willing to travel from their home addresses. 

Prior to data collection, store owners and managers were mailed letters explaining the purpose of the study and giving them the option to decline participation. One store refused to participate, so our final sample consisted of 24 stores. The Institutional Review Board at the University of Illinois designated the procedures for this study as exempt.

### 2.3. Audit Instrument and Data Collection Procedures

Between November 2011 and April 2012, a validated food store audit tool (interrater reliability = 0.81) was used to examine the types of marketing claims (e.g., nutrition, convenience, and taste), the frequency of these claims, and the techniques used (e.g., inclusion of cartoon characters) to market 114 different items sold in our sample of 24 retail food stores. The food items were selected using competitive media reports and a literature review of items commonly advertised to US youth on television and the Internet [36]. The final list of items was sorted into 12 categories as follows: fruits and vegetables, dairy (e.g., milk and flavored milk products), bread and pastries, candy and gum, breakfast cereal, cookies and crackers, juice, other snacks (e.g., Jell-O snacks), prepared foods (e.g., Kraft Macaroni and Cheese), salty snacks (e.g., Doritos chips), and soda (the audit instrument and list of food items may be requested from the lead author). 

### 2.4. Measures

**Figure 1:**
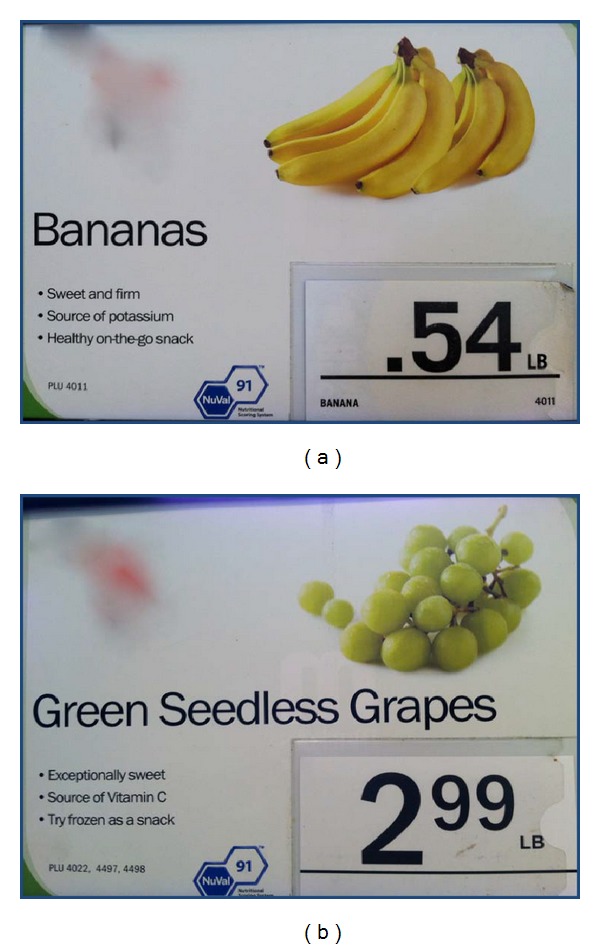
In-store marketing for bananas and grapes showing health and nutrition related claims and NuVal score. NuVal is a nutritional scoring system that allows consumers to assess the nutritional value of foods with a score between 1 and 100. Foods with higher values are considered to have better nutrition.

**Figure 2:**
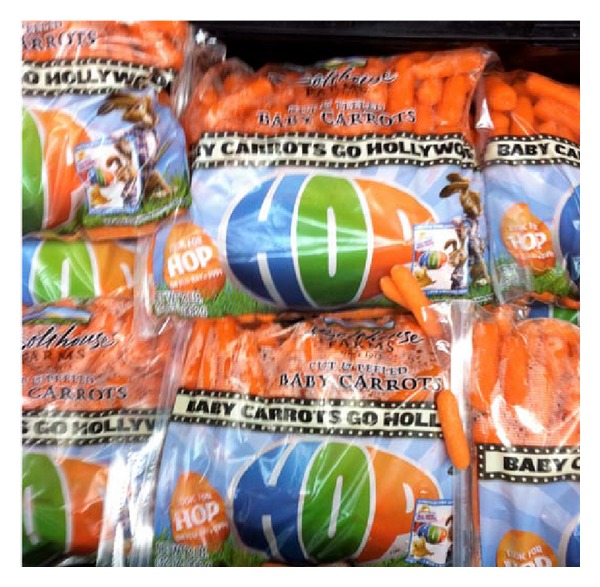
Movie tie-in for carrots using the movie *HOP, *a 2011 live action/animated family film.

**Figure 3:**
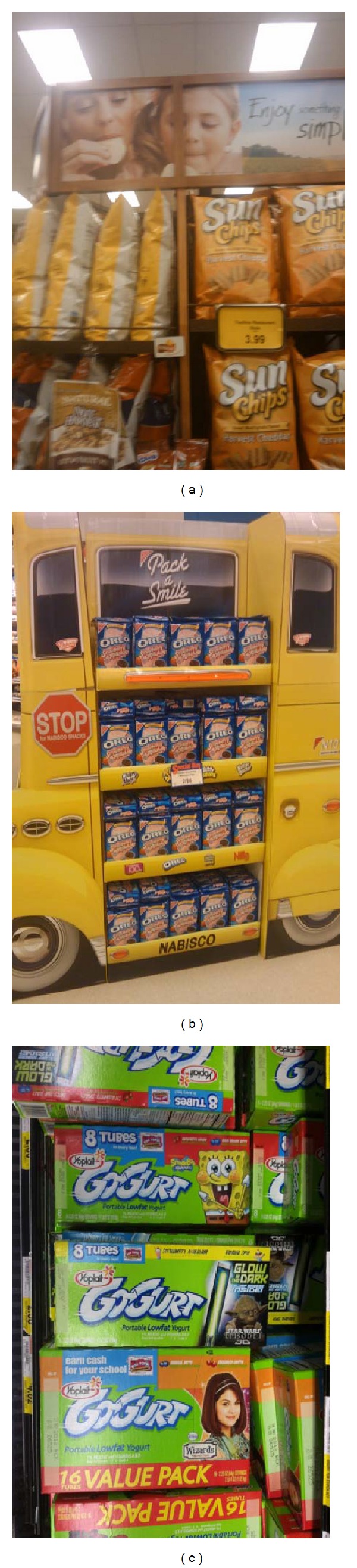
In-store marketing for packaged items including (left to right) chips, cookies, and yogurt.

We defined *availability* as the presence or absence of each of the 114 food items. The fruits and vegetables selected were based on commonly consumed items in the United States [37], as well as supermarket marketing campaigns for fruits and vegetables by the Walt Disney company [38]. Marketing techniques were evaluated by assessing the packaging on food items for the presence of cartoon or spokes-characters (e.g., Dora the Explorer, Tony the Tiger), nutrition claims (no trans fat), and tie-ins for children's movies and television shows. In addition, packaging was assessed for taste claims (flavor blasted), statements regarding convenience (“ready-to-eat”), fun (“made for fun”), suggested use (add to salad), and promises of toys inside, or information about prize or merchandise giveaways (Table 1). Figures 1, 2, and 3 provide examples of marketing techniques that we observed in stores included in our sample.

### 2.5. Data Analysis

We used chi-square tests, or the Fisher exact test for small cell sizes, to determine whether significant differences existed in the marketing of items by store type (grocery versus convenience store), store food-assistance-program acceptance (WIC versus non-WIC), and claims. Stores that had fresh produce and meat sections were classified as grocery stores, and those with either fresh produce or meat sections were classified as convenience stores for data analysis. We calculated a marketing-to-availability (*M* : *A*) ratio to determine how often any of the 8 marketing techniques were used when an item was available. Chi-square tests were used to test for differences in the *M* : *A* ratio between store types and food assistance programs. SPSS version 20 was used to run all analyses [39].

## 3. Results 

Table 2 summarizes our observations of availability and marketing of youth-targeted foods in the stores in our study. Across the 24 food stores, salty snacks and soda had the highest in-store overall claim frequency (83.33%), while fruits and vegetables had the lowest frequency (33.33%). 

WIC stores were more likely to carry items that had some form of marketing, while items surveyed in non-WIC stores exhibited less marketing overall, with fruits and vegetables and other snacks presenting the fewest marketing claims or techniques (e.g., television tie-ins). All of the WIC stores we surveyed carried soda and juice, but only 63.60% carried fruits and vegetables. Using the Fisher exact test, we found statistically significant differences between WIC and non-WIC store claim frequency for 8 of our 12 categories: fruits and vegetables, dairy, bread and pastries, cereal, juice, other snacks, peanut butter and jelly, and prepared foods. 

Overall, convenience stores were more likely to have marketing claims for available items compared to grocery stores, with statistically significant results found for marketing claims in all food-item categories except other snacks. Grocery stores were more likely to have marketing for fruits and vegetables than convenience stores. For dairy products, however, this was reversed, with convenience stores providing more dairy items with marketing claims than grocery stores. Convenience stores also had more marketing claims for bread and pastries, candy, cookies and crackers, juice, salty snacks, and soda. 

Table 3 summarizes the *M* : *A* ratio for all items included in our study. Of our 12 categories, these 9 always carried some type of marketing claim if they were available in a store: dairy products, bread and pastries, candy, cereal, cookies and crackers, other snacks, prepared foods, salty snacks, and soda. Fruits and vegetables had the lowest *M* : *A* ratio at 57.14%. 

Nutrition claims were found on all available dairy, candy, cereal, salty snacks, and soda but were observed on available fruits and vegetables only half the time (49.99%). Available dairy products were least likely to have marketing for taste or fun, and fruits and vegetables were least likely to have claims for convenience or fun. Bread and pastries, prepared foods, and salty snacks were most likely to have fun claims. Bread and pastries, candy, and cookies and crackers were most likely to have suggested use claims. Convenience claims were the least often observed across all items but were most often found on fruits and vegetables, other snacks, prepared foods, and salty snacks. The only significant difference between the groups of items was for any claim (*P* = .001); that is, no significant differences were found for any of the specific marketing claims.

Table 4 summarizes the *M* : *A* ratio for movie tie-ins, television tie-ins, and cartoons, and the inclusion of toys or giveaways for items evaluated. Overall, cartoon or spokes-characters (69.16%) were used most often as a marketing technique when these items were available, followed by the inclusion of toys or giveaways, television tie-ins, and movie tie-ins in descending order. Cartoons were used most often with candy and least often with soda. Dairy products were also found to make substantial use of cartoons in marketing (83.33%). Toys or giveaways were used most often with other snacks and breakfast cereal and not at all with fruits and vegetables or peanut butter and jelly products. Television tie-ins were used most often with breakfast cereals, while movie tie-ins were used most often for prepared foods and dairy products. We did not find any significant differences for marketing techniques between the groups of items.

When we compared the *M* : *A* ratio of the stores where mothers actually shopped to all of the stores examined in a previous study in the study area [36], we found that more marketing existed in the stores that mothers identified as their primary stores of choice for the following 6 categories: dairy products (75% versus 55.5%), bread and pastries (75% versus 25.8%), candy (79.16%), cereal (70.83% versus 36.1%), prepared foods (66.6% versus 49.5%), and soda (83.3% versus 62.2%). 

## 4. Discussion

Consistent with previous studies [15, 25, 36, 40], most stores in our sample were more likely to have marketing claims for less healthy food products, such as salty snacks, than for typically healthier fare, such as fruits and vegetables. With regard to convenience stores as a subcategory, the convenience stores included in our sample were more likely to have marketing than the convenience stores sampled in an earlier study by Grigsby-Toussaint et al. [36]. The difference may be because we focused specifically on stores identified by mothers of young children, while the earlier study targeted all stores in the study area [36]. Results from the current sample suggest that families with young children may actually be exposed to more food marketing within the retail food store environment than previously thought. This finding is critical as we continue studying the impact of the food environment on obesity risk among children and families and suggests that it may be better to examine the stores where parents actually shop rather than attempting to survey all food stores in a particular area. 

Although many products attempt to convey a “health halo” with claims for nutrition [41], barely 50% of available fruits and vegetables in our study included any nutrition claims at all. We did find, however, that packaged fruits and vegetables, such as bags of salad or apples, often featured nutrition claims, so perhaps additional efforts are needed to sell prepared fruits and vegetables (e.g., salads) as single serving packaged items that make it easier to market in the retail food store. In fact, this suggestion is aligned with recent research supported by the Produce for Better Health Foundation that examined strategies to encourage increased fruit and vegetable consumption [42]. The fact that marketing expenditures on fruits and vegetables are limited may provide an opportunity to encourage companies to spend more and improve marketing for fruits and vegetables as healthier snacks for kids, especially highlighting claims for convenience, taste, and fun. 

Although all dairy products carried claims for nutrition, there is clearly an opportunity to improve claims for convenience and suggested use because milk can be sold as a single serving (convenience) and also as part of (fun) healthy snacks (suggested use). In fact, this approach may already be having an effect: increased marketing for dairy products between 2006 and 2009 (from $54 million to $78 million) was shown to result in a moderate increase in dairy consumption, primarily for yogurt products [18].

The high prevalence of cartoon and spokes-characters—69% across the majority of the items assessed—suggests the desirability of restricting marketing to less healthful items or, conversely, the desirability of encouraging the use of these characters on more healthful items such as fruits and vegetables. Only 36% of fruits and vegetables used a cartoon or spokes-character, but we think that this marketing technique has the potential to capture the attention of children and families in the retail food store environment.

This study has several limitations. First, the study was cross-sectional and limited to the winter and spring months, which may explain the limited availability and marketing of fruits and vegetables in our sample; that is, our study does not account for the impact of seasonality on fruit and vegetable availability. Second, we did not collect data on pricing, which could also influence purchasing practices. Third, receipts were not used to validate the potential impact of marketing of specific items on the purchases of mothers of young children in our study area. Fourth, movie tie-ins may generally be more prevalent over the summer months when film studios introduce more movies for the general public, although our study period did capture both the Thanksgiving and Christmas holidays when ample family fare is also introduced in the North American movie market [43]. Finally, although we used a list of items based on marketing typically targeting the youth via television and the Internet, some of the stores in our sample were international grocery stores that did not always carry all of the products on our list. This might explain lower levels of marketing for items such as dairy products and breakfast cereals in grocery stores compared to convenience stores.

Despite these limitations, our study has several strengths. First, our study represents one of the few examinations of the food store environment that focuses specifically on stores where mothers actually shop for their children and families. Second, our sampling frame accounts for varying levels of socioeconomic status by including women who participate in the WIC program. Third, our study had a very high response rate (96%), with only one store refusing an audit. 

A recent analysis by the United States Department of Agriculture (USDA) showed the increased use of health and nutrition-related claims (HNR) by food and beverage companies [44]. This increase is hypothesized to be associated with a growing interest in the perceived benefits of improving lifestyle habits related to dietary intake [44]. Although more healthful items such as fruits and vegetables showed an increase in HNR, a decrease in HNR for less healthful items such as snacks and desserts was found [44]. In our sample of stores, however, we found that fruits and vegetables had the lowest overall frequency of HNR, while snacks had one of the highest overall frequencies. Our finding indicates that stores where mothers actually shop are critically important for interventions, as they not only seem to have more marketing for less healthful foods but also ensure that we reach our population of interest. Retail food stores provide an important setting for population-based interventions using food marketing to encourage healthy dietary behaviors among children and families. The availability and marketing of more healthful items, such as fruits and vegetables, might encourage mothers and caregivers of young children to make healthier choices, thus potentially diluting the marketing effects of less healthful food items.

## Figures and Tables

**Table 1:** Claim examples.

Claim type	Examples	Food type
Nutrition	Low-fat snack	Candy
	Can help lower cholesterol	Cereal
	38% less fat	Dairy
	No sugar added	Other snacks
	No trans fat	Salty snacks

Taste	Long-lasting flavor	Candy
	Rocks your mouth	Cereal
	Flavor blasted	Cookies and crackers
	Famous taste	Prepared foods
	Bursting with 35% more flavor	Salty snacks
	Crisp, clean, and refreshing	Soda

Fun	Made for fun	Breads and pastries
	A little fun any time	Candy
	Joy of snacking	Cereal
	Great tasting fun	Juice
	Fuel the fun	Peanut butter and jelly

Suggested use	Freeze n' go	Dairy
	Add to salad	Fruits and vegetables
	Perfect snack	Fruits and vegetables

Convenience	Two to go—save one for later	Candy
	To go	Cookies and crackers
	Ready to eat	Fruits and vegetables
	Just heat and enjoy	Prepared foods

**Table 2:** Frequency of Champaign-Urbana food stores (*N* = 24) with item (*N* = 114) claims.

Item	Sample stores (%) (*N* = 24)	WIC and non-WIC store claim frequency (*N* = 24)			Grocery and convenience store claim frequency (*N* = 24)		
		WIC (%) (*n* = 11)	Non-WIC (%) (*n* = 13)	*P* value^a^	Grocery (%) (*n* = 12)	Convenience (%) (*n* = 12)	*P* value^a^
Fruits and vegetables	33.33	63.63	7.69	.006	58.33	8.33	.02
Dairy	75.00	81.82	69.23	.04	66.66	83.33	.002
Bread and pastries	75.00	90.91	61.53	.005	50.00	100.00	<.001
Candy	79.16	90.91	69.23	.64	58.33	100.00	.07

Cereal	70.83	81.82	61.53	.003	50.00	91.66	<.001
Cookies and crackers	75.00	90.91	61.53	.14	50.00	100.00	<.001
Juice	62.50	100.00	30.83	<.001	58.33	66.66	.03
Other snacks	41.66	81.82	7.69	<.001	50.00	33.33	.19

Peanut butter and jelly	45.83	81.82	15.38	.002	50.00	41.66	.01
Prepared foods	66.66	90.91	46.16	.002	50.00	83.33	.01
Salty snacks	83.33	90.91	76.92	.54	66.66	100.00	.03
Soda	83.33	100.00	69.23	.06	66.66	91.66	.02

Total	65.97	87.12	48.11		56.24	75.00	

WIC: Supplemental Nutrition Program for Women, Infants, and Children.

^a^Fisher exact test.

**Table 3:** Marketing-to-availability ratio of Champaign-Urbana (*N* = 24) food stores carrying items (*N* = 114) with nutrition, taste, fun, suggested use, or convenience claims.

Item	Claim (%)*	Nutrition (%)	Taste (%)	Fun (%)	Suggested use (%)	Convenience (%)
Fruits and vegetable	57.14	49.99	42.85	7.13	57.14	28.56
Dairy	100.00	100.00	22.21	5.54	38.88	16.66
Bread and pastries	100.00	94.44	88.88	88.88	100.00	5.54
Candy	100.00	100.00	94.74	73.68	94.74	5.25

Cereal	100.00	100.00	94.11	41.16	41.16	29.40
Cookies and crackers	100.00	94.44	100.00	83.33	100.00	27.77
Juice	75.00	75.00	60.00	19.99	30.00	4.99
Other snacks	100.00	100.00	50.00	19.99	50.00	30.00

Peanut butter and jelly	78.57	64.28	35.71	64.28	21.42	0.00
Prepared foods	100.00	93.75	87.50	87.50	93.75	37.50
Salty snacks	100.00	100.00	100.00	94.99	49.99	34.99
Soda	100.00	100.00	90.00	9.99	4.99	0.00

Total	92.56	89.33	72.17	49.71	56.84	18.39

*P < .001.

**Table 4:** Marketing-to-availability ratio of movie, TV, cartoon, and toy giveaways on food items.

Food items	Movie (%)	TV (%)	Cartoon (%)	Toy giveaways (%)	Total (%)
Fruits and vegetables	7.13	0.00	35.71	0.00	10.71
Dairy	16.67	22.23	83.33	33.33	38.89
Bread and pastries	11.11	0.00	94.44	11.11	29.17
Candy	5.25	5.25	100.00	36.83	36.83

Cereal	11.76	52.94	94.11	52.94	52.94
Cookies and crackers	0.00	0.00	94.44	16.67	27.78
Juice	0.00	0.00	46.66	6.66	13.33
Other snacks	0.00	30.00	69.98	69.98	42.49

Peanut butter and jelly	0.00	0.00	49.99	0.00	12.50
Prepared foods	25.00	25.00	56.25	50.00	39.06
Salty snacks	0.00	0.00	90.00	4.99	23.75
Soda	4.99	25.00	15.00	25.00	17.50

Total	6.83	13.37	69.16	25.63	
